# Aging and drug discovery

**DOI:** 10.18632/aging.101646

**Published:** 2018-11-13

**Authors:** Daniela Bakula, Alexander M. Aliper, Polina Mamoshina, Michael A. Petr, Amanuel Teklu, Joseph A. Baur, Judith Campisi, Collin Y. Ewald, Anastasia Georgievskaya, Vadim N. Gladyshev, Olga Kovalchuk, Dudley W. Lamming, Martijn S. Luijsterburg, Alejandro Martín-Montalvo, Stuart Maudsley, Garik V. Mkrtchyan, Alexey Moskalev, S. Jay Olshansky, Ivan V. Ozerov, Alexander Pickett, Michael Ristow, Alex Zhavoronkov, Morten Scheibye-Knudsen

**Affiliations:** 1 Center for Healthy Aging, Department of Cellular and Molecular Medicine, University of Copenhagen, Copenhagen, Denmark; 2 Pharmaceutical Artificial Intelligence Department, Insilico Medicine, Inc., Baltimore, MD 20850, USA; 3 Institute for Diabetes, Obesity and Metabolism, Perelman School of Medicine, University of Pennsylvania, Philadelphia, PA 19104, USA; 4 Department of Physiology, Perelman School of Medicine, University of Pennsylvania, Philadelphia, PA 19104, USA; 5 Buck Institute for Research on Aging, Novato, CA 94945, USA; 6 Lawrence Berkeley National Laboratory, Life Sciences Division, Berkeley, CA 94720, USA; 7 Institute of Translational Medicine, Department of Health Sciences and Technology, Swiss Federal Institute for Technology (ETH) Zürich, Switzerland; 8 Youth Laboratories, Moscow, Russia; 9 Division of Genetics, Department of Medicine, Harvard Medical School, Brigham and Women's Hospital, Boston, MA 02115, USA; 10 Belozersky Institute of Physico-Chemical Biology, Moscow State University, Moscow, Russia; 11 Department of Biological Sciences, University of Lethbridge, Lethbridge, Alberta, Canada; 12 Pathway Rx Ltd, Lethbridge, Alberta, Canada; 13 Department of Medicine, University of Wisconsin-Madison, Madison, WI 53706, USA; 14 Department of Human Genetics, Leiden University Medical Center, Leiden, The Netherlands; 15 Pancreatic Islet Development and Regeneration Unit/Laboratory of Aging Biology, Centro Andaluz de Biología Molecular y Medicina Regenerativa-CABIMER, Universidad de Sevilla-CSIC-Universidad Pablo de Olavide, Seville, Spain; 16 Department of Biomedical Sciences, University of Antwerp, Antwerp, Belgium; 17 Translational Neurobiology Group, Center of Molecular Neurology, VIB, Antwerp, Belgium; 18 Engelhardt Institute of Molecular Biology, Russian Academy of Sciences, Moscow, Russia; 19 Institute of Biology of Komi Science Center of Ural Branch of RAS, Syktyvkar, Russia; 20 Moscow Institute of Physics and Technology, Dolgoprudny, Russia; 21 Division of Epidemiology and Biostatistics, University of Illinois at Chicago School of Public Health, Chicago, IL 60612 USA; 22 Juvenescence Limited, Douglas, Isle of Man, UK

**Keywords:** aging, drug discovery

## Abstract

Multiple interventions in the aging process have been discovered to extend the healthspan of model organisms. Both industry and academia are therefore exploring possible transformative molecules that target aging and age-associated diseases. In this overview, we summarize the presented talks and discussion points of the 5th Annual Aging and Drug Discovery Forum 2018 in Basel, Switzerland. Here academia and industry came together, to discuss the latest progress and issues in aging research. The meeting covered talks about the mechanistic cause of aging, how longevity signatures may be highly conserved, emerging biomarkers of aging, possible interventions in the aging process and the use of artificial intelligence for aging research and drug discovery. Importantly, a consensus is emerging both in industry and academia, that molecules able to intervene in the aging process may contain the potential to transform both societies and healthcare.

## Introduction

“Why do we age?”; “Can we intervene in the aging process?”; and if so, what approaches should aging science take to transform research into viable therapeutic interventions to improve public health? Understanding the mechanisms of aging will be of vital importance to answering these questions. However, several obstacles stand in the way of generating efficacious and safe interventions that extend the period of healthy life.

At the 5^th^ Annual Aging and Drug Discovery Forum which was held during the Basel Life Congress, Basel, Switzerland, September 12-13, 2018, leading aging experts from academia and industry came together to discuss top issues in aging research. The meeting was organized by Alex Zhavoronkov, CEO of Insilico Medicine (Baltimore, MD, United States), a company specialized in implementing artificial intelligence (AI) solutions for drug discovery and biomarker development and Morten Scheibye-Knudsen, head of the aging interventions lab at the Center for Healthy Aging, University of Copenhagen (Copenhagen, Denmark). Here, we provide a brief overview of the presented results and discussion points.

### What drives the aging process?

Aging is a multifactorial process that leads to loss of cellular homeostasis followed by susceptibility to diseases. The functional decline is influenced by highly conserved signaling pathways that could be targeted to manipulate the aging process. **Judith Campisi** from the Buck Institute for Research on Aging (Novato, CA, United States) highlighted the impact of cellular senescence as an evolutionary balancing act on aging [1]. Senescent cells accumulate over time and promote the aging processes by loss of tissue functionality and through the secretion of pro-inflammatory factors, known as the secretory phenotype [2]. Senescence represents a double-edged sword by repressing malignant transformation. However, accumulation over time causes degenerative phenotypes and drives tumor formation. Therefore, targeting senescent cells using senolytics or by compensating for the beneficial effects of the induced secretory phenotype, may help to prevent age-dependent functional decline. Clear markers for senescent cells are still lacking, due to the strong heterogeneity of senescent cells [3,4]. To address this issue, the group of Judith Campisi implemented single cell analysis to further understand the cell-type specific signatures of senescent cells, which may help to find more specific targets [4].

**Michael Ristow** from the ETH Zürich (Zürich, Switzerland) presented his work about redox-regulation of lifespan and metabolism. Previous results of his group suggest the concept of mitohormesis as a health-promoting signaling pathway, where low concentrations of reactive oxygen species (ROS) lead to an adaptive response [5]. The concept of mitohormesis was further supported by the observation that glucose restriction in *C. elegans* leads to increased ROS production, thereby promoting endogenous stress defense and increased lifespan [6]. ROS-induced increased insulin sensitivity was also observed in a physical exercise trial in humans and, interestingly, this effect was inhibited by the supplementation of antioxidants [7]. Furthermore, his group compared gene expression levels of young, mature and old *C. elegans*, *D. rerio* and *M. musculus* to predict evolutionary conserved age-related genes. Branched-chain amino acid transferase 1 (*bcat-1*) was identified as a strong lifespan regulator and, accordingly, knockdown of *bcat-1* increased lifespan in *C. elegans* [8].

Both endogenous stress factors, such as ROS and exogenous stress factors are constantly challenging the human genome. Different DNA repair pathways have evolved to repair the different DNA lesions. Indeed, DNA damage accumulate with age and DNA repair capacity has been suggested to decline with age [9]. The impact of DNA repair on aging was emphasized by two presentations at the meeting. **Morten Scheibye-Knudsen** from the University of Copenhagen (Copenhagen, Denmark) discussed the phenotypical landscape of aging [10] and emphasized that the mechanisms causing the different pathologies of aging are poorly understood. Morten Scheibye-Knudsen presented the value of using hierarchical clustering and machine learning algorithms to compare similarities between diseases based on their clinical features [11]. This approach may help to find cellular pathways that are linked to specific disease pathologies. Notably, diseases caused by defects in DNA repair resembles many features of aging, emphasizing genome maintenance as a key factor of aging [12]. Thus, premature aging disorders may be good model systems to study aging.

This observation was underscored by **Martijn Luijsterburg** from the Leiden University Medical Center (Leiden, The Netherlands) who presented his latest research on transcription-coupled DNA repair, a DNA repair pathway that removes lesions blocking transcription of active genes. Interestingly, mutations in transcription-coupled repair genes are known to cause diverse phenotypes [13]. Mutations in CSA and CSB can cause the premature aging disorder Cockayne syndrome which is characterized by severe neurodegeneration, whereas mutations in UVSSA can cause the UV-sensitivity syndrome, a disease without neurological phenotypes. Untangling the precise molecular functions of the transcription coupled repair complex members may help to understand the disease mechanism of Cockayne syndrome and aging features. Martijn Luijsterburg presented his latest results towards this understanding.

### Signatures of longevity

In recent years, a concerted effort was made to identify biological markers that can predict the chronological age of an individual. Different types of age predictors including telomere length [14], gene expression changes [15] and epigenetic changes, the latter known as the epigenetic clock [16–18], have been shown to strongly correlate with chronological aging. **Vadim Gladyshev** from the Brigham and Women’s Hospital, Harvard Medical School (Boston, MA, United States) and Moscow State University (Moscow, Russia) highlighted that the combination of different approaches can allow for more reliable biological age prediction, since aging is a systemic process. His research group investigated the mouse blood DNA methylome of different age groups to develop an epigenetic clock that can be used to test aging interventions [19]. Notably, this analysis showed that caloric restriction, as well as the long-lived mutant Snell dwarf mice, exhibit slower epigenetic aging. A further study of his group determined longevity-related transcriptomic changes across 33 species of mammals [20] and 14 different Drosophila species [21], which may be used for prediction of new longevity interventions.

**Collin Ewald** from the ETH Zürich (Zürich, Switzerland) presented his recent work on the transcriptomic signatures of longevity in *C. elegans*, revealing that the extracellular matrix (ECM) composition undergo changes with age. Notably, potential aging interventions, such as rapamycin treatment, modulate ECM gene expression [22]. Moreover, reduced insulin/IGF-1 signaling induced lifespan extension was shown to be dependent on collagen gene expression. These observations indicate that modulation of the ECM may be a promising target. Collin Ewald’s work reveals the value of C. elegans as a powerful model system to study aging and aging interventions, due to its relatively short life span and easily manipulated genome.

In addition to epigenetic and gene expression changes, profound age-dependent remodeling of protein expression can be observed with age [23]. **Stuart Maudsley** from the University of Antwerp (Antwerp, Belgium) discussed the identification of multidimensional regulators of aging. In several recent studies, his group has examined, using a combination of classical signaling pathway analysis and natural language processing informatics, age-related proteomic alterations in the hypothalamus, the potential key organ that coordinates global somatic aging [24]. Using this novel approach, the G protein-coupled receptor kinase interacting protein 2 (GIT2) was identified as a potential key regulator of aging. Consequently, GIT2 protein expression was demonstrated to be strongly age-sensitive in the hypothalamus and multiple other brain regions as well as a broad range of peripheral tissues associated with energy metabolism regulation [24,25]. GIT2 acts a scaffold protein for multiple signaling pathways, hence decreased GIT2 function affects several hallmarks of aging including metabolic dysfunction [25], DNA damage responses [26], oxidative damage responsiveness [27] and immune system senescence [28]. Thus, GIT2 may be a promising multidimensional therapeutic target for age-associated diseases [29,30].

### Interventions in aging and aging-associated disease

In the last years multiple interventions for life span extension have been identified. Pharmacological (e.g. rapamycin, resveratrol, metformin) and non-pharmacological interventions (e.g. caloric restriction, physical exercise) have been extensively discussed. Nevertheless, how these interventions may affect human health span is not well understood. A growing number of studies suggest that the macronutrient composition of the diet, and in particular dietary protein, may also play a crucial role in regulating metabolic health and life span [31]. However, it remains an open question as whether altered consumption of specific dietary amino acids mediates the beneficial effects of reduced dietary protein. In this regard, **Dudley Lamming** from the University of Wisconsin (Madison, WI, United States) presented his work about promoting healthy aging through the reduction of specific dietary macronutrients. Dudley Lamming’s research group provided data revealing that the reduction of branched chain amino acids improved metabolic health in lean as well as diet-induced obese C57BL/6J mice, and recapitulated many of the metabolic benefits of a low protein diet [32,33]. Notably, a reduction of leucine alone stimulated white adipose tissue growth, indicating that individual branched-chain amino acids may have distinct roles in the regulation of metabolic health [33].

Due to the difficulty to reduce caloric intake, compounds mimicking the positive effects of caloric restriction may be a promising strategy, as outlined by **Joseph Baur** from the University of Pennsylvania (Philadelphia, PA, United States). Modulating nicotinamide adenine dinucleotide (NAD^+^) levels is a promising strategy to treat age-associated physiological decline [34,35]. Recent work from Joseph Baur revealed that muscle specific knockout of nicotinamide phosphoribosyltransferase (NAMPT), an enzyme that is essential for the maintenance of normal NAD^+^ concentration, lead to progressive loss of muscle function. The observed phenotype was counteracted by administration of the NAD^+^ precursor nicotinamide riboside [36]. Interestingly, lifelong NAMPT overexpression prevented the age-related loss of NAD^+^ and improved exercise performance in old mice. Although mitochondrial NAD^+^ pools have been shown to contribute to cell metabolism, the source of mitochondrial NAD^+^ remains an obscurity. A recently published study by the group of Joseph Baur proposes the existence of a so far undiscovered mitochondrial NAD^+^ transporter, which may be a further potential target to modulate the compartmentalization of NAD^+^ within the cell [37].

**Alexey Moskalev** from the Moscow Institute of Physics and Technology (Moscow, Russia) emphasized the potential of natural compounds as aging interventions. Alexey Moskalev presented data about a potential geroprotective function of the carotenoid fucoxanthin in *C. elegans* and *D. melanogaster* [38]. Fucoxanthin increased the median lifespan of *C. elegans* and *D. melanogaster*, along with changes associated with aging such as increased stress resistance. At the molecular level the response to fucoxanthin in *D. melanogaster* goes along with transcriptional changes of longevity-related signaling pathways, such as the MAPK, mTOR and autophagy pathways [39]. Preliminary data on human fibroblasts shows antisenogenic effects of fucoxanthin at 100 nM and 1 nkM (unpublished).

**Olga Kovalchuk** from the University of Lethbridge and PathwayRx Inc. (Lethbridge, Canada) gave an overview of skin aging driven by intrinsic and extrinsic factors leading to deregulated matrix metalloproteinases (MMPs), enzymes activated by UV exposure, inflammation and other factors [40]. MMPs contribute to the breakdown of collagen while inhibiting new collagen formation, and cytokine deregulation could be possible intervention targets by novel extracts. She gave an overview of the effects of UV and THz radiation on skin, and presented novel (patent pending) plant extracts with very potent anti-inflammatory and anti-aging capacities that can be used to counteract harmful environmental effects of skin and potentially reverse skin aging.

**Alejandro Martín-Montalvo** from the Andalusian Molecular Biology and Regenerative Medicine Centre -CABIMER- (Seville, Spain) discussed the potential of interventions based on the use of thyroid hormones on aging and age-associated diseases. The key role of thyroid hormone levels in regulating longevity is corroborated by the observation, that humans with exceptional longevity, and other long-lived animals, show decreased circulating thyroid hormone levels [41]. Nevertheless, Alejandro Martín-Montalvo showed that thyroid hormones enhance glucose clearance, leading him to investigate if these hormones could be used for the treatment of different types of diabetes. Indeed, his team has shown, using two experimental models of diabetes, that thyroid hormones efficiently blunt the onset of diabetes and increase survival in mice [42]. The data indicate a potential benefit of thyroid hormones and/or thyromimetics for the treatment of type 1 diabetes mellitus.

### The power of AI to facilitate anti-aging drug discovery

In comparison to conventional drug development, the development and commercialization of pharmaceuticals targeting aging pose additional difficulties. **Alexander Pickett** (Juvenescence Ltd, Boston, MA, United States), SVP of Business Development at Juvenescence Limited, a drug development company focusing on therapeutics that modify aging, highlighted the obstacles to developing aging interventions. The lack of reliable techniques to prove the efficacy of the therapeutics is one of the main difficulties of clinical trial designs for aging interventions. In this regard, Alexander Pickett pointed out the importance of developing new reliable biomarkers and validating that they were not only predictive of aging but also responded to known interventions. Another obstacle will be commercializing aging intervention drugs: so far, our health care systems rely predominantly on treating illness rather than keeping people healthy. Juvenescence believes that developing aging interventions to treat existing diseases and seeking outcomes-based reimbursement is the quickest path to bringing aging interventions to large populations. To address these issues, Juvenescence Limited has invested in several biotech startups that pursue a whole range of approaches, including Insilico Medicine.

**Ivan Ozerov** from Insilico Medicine (Baltimore, MD, United States) introduced an AI-driven computational pipeline approach to identify new small molecules that counteract the progression of cellular senescence. The approach relies on the concept of 5R (Rescue, Remove, Replenish, Reinforce, Repeat) and utilize the recently published in silico Pathway Activation Network Decomposition Analysis (iPANDA) method [43]. The pipeline may accelerate target and drug discovery for anti-aging interventions.

The power of Artificial Intelligence to facilitate drug discovery was highlighted further by **Garik Mkrtchyan** from the Scheibye-Knudsen lab (Copenhagen University, Denmark). Increased unrepaired DNA lesions are a hallmark of aging. To counteract this, stimulating DNA repair may be a promising approach. Garik Mkrtchyan presented work about deep learning algorithms utilized to screen a small molecule library to identify compounds that stimulate DNA repair. The success of the approach was proven by the identification of drugs that induced cellular resistance to ionizing radiation but do not cause DNA damage (unpublished data).

**S. Jay Olshansky** from the University of Illinois (Chicago) and co-founder of Lapetus Solutions, Inc., further emphasized the requirement of biomarkers to facilitate the identification of aging interventions. He made a point that the target of intervention should be healthspan and not lifespan [44]. Olshansky showed that biological age is reflected in facial features and underlined the value of photographic phenotypic biomarkers in health assessment. Based on facial photographs, age and gender can be predicted with a high degree of reliability, but also further risk factors such as smoking behavior and body mass index can be revealed, which makes it relevant to insurance and financial sectors as well. Facial analytics as a promising biomarker for aging, was further acknowledged by **Anastasia Georgievskaya**, a co-founder of Youth Laboratories (Moscow, Russian Federation). Youth Laboratories is using artificial intelligence to study aging and discover new class of non-invasive photographic aging biomarkers. Currently, the company is collecting photographs of lab mice to develop the first photographic biomarker for mice. Since mice are still the main model of aging research, the photographic mouse aging clock may facilitate the identification of beneficial aging interventions in mice to further translate them to humans.

### Panel discussion on key trends in longevity biotechnology

The panel discussion on longevity biotechnology industry with Vadim N. Gladyshev, Morten Scheibye-Knudsen, Judith Campisi, Alexander Pickett, Michael Antonov, Joseph A. Baur and Stuart Maudsley and chaired by Alex Zhavoronkov, identified the following areas with the most potential of commercialization: repurposing of drugs that are already on the market, senolytics, mTOR inhibitors, NAD^+^ activators and modulators, reprogramming-based and regenerative-based approaches, AI and data analysis, and digital health.

During the discussion, the panel highlighted the great potential of drug repurposing (repositioning) for aging research. Drug repurposing or target extension allows the identification of new indications for drugs with low safety risk profiles, as they have been already tested in humans. Great progress has been made in developing approaches for drug characterization and classification that could be used for drug repurposing [45–47]. Indeed, several attempts have been made to predict a potential aging application of existing medications [48–52].

Recently, several senolytics, selective inductors of senescence cell death, have been tested as aging preventive or aging revertive agents [53,54]. At the same time, the panel emphasized that such therapies have high-risk profiles, as they are only in early phases of clinical development [55]. mTOR inhibitors [56] and NAD^+^ activators [57,58] have also been listed as interventions that could delay the onset of multiple age-related pathologies. Therefore, they could be explored as human aging therapies in the nearest future. One panelist made the point that regenerative medicine approaches [59] could provide therapeutic possibilities for targeting aging and could be developed further to reach the market.

AI and deep learning are now recognized as transformative technologies in healthcare in general [47,60,61]. For instance, a lot of progress has been made in machine-learning-based biomarkers of human aging using easily obtained data such as methylation [16,17], transcriptomics [62], proteomics [63] and blood biochemistry [64,65]. Following an extensive discussion, panelists selected AI and digital health as key developments that would catalyze the pace of innovation in aging research. All panelists agreed that aging research and longevity interventions have tremendous opportunities to impact the whole healthcare sector.

### Closing remarks

Clearly, aging interventions require a multifaceted approach through which complex phenotypes can be evaluated using next generation machine learning approaches. The meeting underscored the awareness that drug discovery in the aging field will require not only determination from academic researchers but also multiple industrial partners and investors. This state of the field is particularly clear in light of the significant number of emerging companies that attempt to tackle the task of finding translatable interventions into aging. It is evident from the formation of all these startups that the next promising longevity pharmaceuticals will be found. The future looks bright.
